# *Rhinacanthus nasutus* “Tea” Infusions and the Medicinal Benefits of the Constituent Phytochemicals

**DOI:** 10.3390/nu12123776

**Published:** 2020-12-09

**Authors:** James Michael Brimson, Mani Iyer Prasanth, Dicson Sheeja Malar, Sirikalaya Brimson, Tewin Tencomnao

**Affiliations:** 1 Age-Related Inflammation and Degeneration Research Unit, Chulalongkorn University, Bangkok 10330, Thailand; james.b@chula.ac.th (J.M.B.); Prasanth.I@chula.ac.th (M.I.P.); Sheeja.M@chula.ac.th (D.S.M.); 2 Department of Clinical Chemistry, Faculty of Allied Health Sciences, Chulalongkorn University, Bangkok 10330, Thailand; 3 Department of Clinical Microscopy, Faculty of Allied Health Sciences, Chulalongkorn University, Bangkok 10330, Thailand; Sirikalaya.J@chula.ac.th

**Keywords:** cancer, neurodegenerative disease, diabetes, infectious disease, natural products, phytochemicals, snake jasmine

## Abstract

*Rhinacanthus nasutus* (L.) Kurz (Acanthaceae) (*Rn*) is an herbaceous shrub native to Thailand and much of South and Southeast Asia. It has several synonyms and local or common names. The root of *Rn* is used in Thai traditional medicine to treat snake bites, and the roots and/or leaves can be made into a balm and applied to the skin for the treatment of skin infections such as ringworm, or they may be brewed to form an infusion for the treatment of inflammatory disorders. *Rn* leaves are available to the public for purchase in the form of “tea bags” as a natural herbal remedy for a long list of disorders, including diabetes, skin diseases (antifungal, ringworm, eczema, scurf, herpes), gastritis, raised blood pressure, improved blood circulation, early-stage tuberculosis antitumor activity, and as an antipyretic. There have been many studies investigating the roles of *Rn* or compounds isolated from the herb regarding diseases such as Alzheimer’s and other neurodegenerative diseases, cancer, diabetes and infection with bacteria, fungi or viruses. There have, however, been no clinical trials to confirm the efficacy of *Rn* in the treatment of any of these disorders, and the safety of these teas over long periods of consumption has never been tested. This review assesses the recent research into the role of *Rn* and its constituent compounds in a range of diseases.

## 1. Introduction

*Rhinacanthus* genus comprising of about 25 species belongs to the family Acanthaceae and distributed throughout tropical and subtropical regions [1]. *Rhinacanthus nasutus* (*Rn*), commonly referred to as snake jasmine because of the shape of its flower, and is a native of Southeast Asia. The plant is also referred to by the names (as well as others (Table 1)) *Rhinacanthus rottlerianus* Nees and *Justicia rottleriana* Wall. It is a small, slender shrub growing to a height of about 0.6 to 1.2 m [2]. It is a woody based plant with sparsely branched club-shaped fruit and four seeds. The whole plant is widely used in traditional medicinal practices for the treatment of diverse disease conditions. Medicinal preparations of the plant in the form of decoctions and herbal tea have been given internally to the people for the treatment of hepatitis, diabetes, hypertension, while the external application in the form of paste has been used by the people who suffer from psoriasis, eczema, ringworm as well as inflammation. Traditionally seeds, roots and leaves of the plant have been used against scabies, eczema and various skin conditions. Leaves are used for prickly heat as well as scurf, and roots are being boiled along with milk and used as an aphrodisiac. A decoction of roots is used as an antidote for snake bites. Stem and leaves of the plant are used to treat pulmonary tuberculosis and high blood pressure [3].

Several reports have revealed the folklore and traditional medicinal practices using *Rn* ranging from antioxidant, antimicrobial to anticancer and neuroprotectant. The rhinacanthin-rich leaf extract has been reported to have antimicrobial activity against *S. mutans, S. epidermidis, P. acnes* and *S. aureus* [4]. Traditional usage of *Rn* in the treatment of skin diseases caused by fungi has been reported, showing that the leaf extract has fungicidal activity by acting on the cell wall causing its degeneration and death [5]. The leaf extract of the plant also showed significant larvicidal activity against *Culex quinquefasciatus, Anopheles stephensi, Aedes aegypti* and prevented the emergence of larvae to adults [6,7]. Further, a formulation containing root extract of the plant has also been prepared for the control of mosquito vectors [8]. Methanolic extract of *Rn* was found to exhibit significant antioxidant activity in cell-free in vitro studies as well could ameliorate oxidative stress and improve the antioxidant status in experimental rats [9,10]. Additionally, methanol extract of the root was reported to exhibit a cytotoxic effect against oral squamous cell carcinoma cells and promyelocytic leukemia cells while showing lower-level cytotoxicity to normal cells [11]. Ethanolic extract of the plant was shown to exhibit hepatoprotective activity against aflatoxin B1 induced toxicity in Wistar rats by enhancing the glutathione level and modulating the activity of serum enzymes [12]. In addition, the root extract inhibited lipopolysaccharides (LPS)-induced inflammation by reducing the level of nitric oxide (NO) in RAW264.7 macrophage cells [11]. Further, *Rn* root extract has also been reported to have a neuroprotective effect against hypoxia as well as glutamate-induced cell death in HT22 cells by bringing back the redox homeostasis [13,14].

Bibliographical analysis of the publications (original research articles) related to *Rn* shows a significant increase in publications from the late 2000s onwards with 11 *Rn* related articles published in 2009, 70 articles in 2015 and 62 so far in 2020. These articles break down into the following subject areas; pharmacology and toxicology (19%), biochemistry, genetics and molecular biology (17%), agricultural and biological sciences (17%), chemistry (14%), medicine (13%), immunology (4%) and others (16%) (data obtained from SCOPUS).

With the recent increase of publications related to *Rn* in mind, this review aims to cover the advances and research into *Rn* over the last decade in the fields of neurodegeneration, cancer, diabetes, as well as *Rn*’s proprieties as an antimicrobial, antiviral and antifungal agent, and assess whether there is enough evidence to warrant the use of *Rn* or compounds isolated from *Rn* in human trails. All plants described in this manuscript have had their names checked at The Plant List [15].

## 2. Phytochemicals Found in *Rhinacanthus nasutus*

*Rn* has been identified as an important medicinal plant due to its medicinal properties for various disease conditions. This has led to the characterizing of the active ingredients responsible for exhibiting these medicinal properties (Table 2). Kodama et al. 1993 has reported the isolation of a naphthopyran derivative 3,4-dihydro-3,3-dimethyl-2H-naphtho[2,3-b]pyran-5,10-dione which also possess antifungal activity [16]. Several phytochemicals including β-amyrin, glutinol, lupeol, stimasterol, sitosterol, umbelliferone, 2-methylanthraquinone, wogonin, oroxylin A, (+)-praeruptorin, p-hydroxy benzaldehyde, methyl vanillate, syringaldehyde, Dehydro-α-lapachone and novel compounds rhinacanthin A-D, G-Q, (RnA-D and RnG-Q) rhinacanthone, 4-acetonyl-3,5-dimethoxy-p-quinol have been isolated by Wu et al. 1998 from the roots, stem and leaves of *Rn* [17,18,19]. Rhinacanthin S (RnS) and RnA isolated from the leaves of *Rn* showed acetylcholinesterase inhibitory activity and cytotoxicity, respectively [20]. RnC has also been found to attenuate renal oxidative stress and protect against streptozotocin-nicotinamide induced diabetic nephropathy in rats [21]. Based on the antiviral property of the plant, several research groups have succeeded in isolating and examining the activity of phytochemicals from the roots and aerial parts of *R. nasutus*. Sendl et al. 1996 has reported the isolation of two naphthoquinones, RnC and RnD, from the whole plant and their antiviral activity against human cytomegalovirus [22]. The lignans Rhinacanthin E and F (RnE and RnF) isolated from the aerial part of the plant have been reported to have significant activity against the influenza virus under in vitro conditions [23]. An antiviral study by Ngoc et al. 2019 with a novel naphthoquinone analog rhinacasutone and seven other known compounds like rhinacanthone, RnC, RnD, RnN, RnQ and RnE and heliobuphthalmin indicated that RnC, RnD, RnN and RnQ exhibit significant antiviral activity against influenza virus A/PR/8/34 (PR8), Anti-Human Rhinovirus 1B (HRV1B), and Coxsackievirus B3 (CVB3) [24]. Kwak et al. 2018 has isolated trans O-coumaric acid, 2,4-dihydroxycinnamic acid, 3,4-dimethoxyphenyl-O-β-D-glucopyranoside, p-hydroxy phenethyl trans-ferulate, 2,3-bis[(4-hydroxy-3,5-dimethoxyphenyl)methyl]-1,4-butanediol,8,8′-bisdihydrosiringenin glucoside, dehydrodiconiferyl alcohol-4-β-D-glucoside and (-)-dehydrodiconiferyl alcohol from the aerial parts of the plant. Among which, 2,3-bis[(4-hydroxy-3,5-dimethoxyphenyl)methyl]-1,4-butanediol and 8,8′-bisdihydrosiringenin glucoside were found to have significant neuraminidase inhibitory activity [25].

## 3. *Rhinacanthus nasutus* and Neurodegenerative Diseases

Neurodegenerative diseases pose a significant health issue since the average ages of the world’s populations are increasing, and there are more older generations alive than at any time in previous decades. Age brings numerous health problems, including neurodegenerative diseases such as Alzheimer’s disease, Parkinson’s disease, multiple sclerosis, and amyotrophic lateral sclerosis. There are currently 44 million people living with Alzheimer’s, 7 million living with Parkinson’s disease worldwide [41,42]. With the rise in population and life expectancy around the world, these numbers are predicted to rise to 76 million by 2030 and 135 million by 2050 for Alzheimer’s disease, and up to 20 million by 2050 for Parkinson’s diseases [42,43].

Treatments for neurodegenerative diseases fall into three general categories; preventative, restorative, and regenerative. The restorative category covers most drugs available for the treatment of neurodegenerative diseases, such as levodopa for Parkinson’s and acetylcholinesterase inhibitors for Alzheimer’s disease. These drugs are often prescribed after symptoms have begun to show, long after the initial neurodegeneration has taken place, with the aim of replacing neurotransmitters that are lost from the neurodegeneration. These treatments often only improve the symptoms but do not slow or reverse the progression of the disease in any way. Regenerative treatments are the holy grail of neurodegenerative diseases, drugs that stimulate the growth of new neurons to replace those that have been lost, thus restoring function or the implantation of stem cells that would grow and differentiate into the desired neuronal cells to replace those that were lost. For now, despite advances in the field, these kinds of drugs and therapies are still a long way from being brought to clinical trials [44]. Preventative treatments tend to cover dietary and behavioral changes that reduce the risk of neurodegenerative diseases. Examples of preventative measures that can reduce the relative risk of developing Alzheimer’s include not smoking, maintaining an active lifestyle, maintaining a healthy weight, and treating diseases such as depression and diabetes.

**Figure 1:**
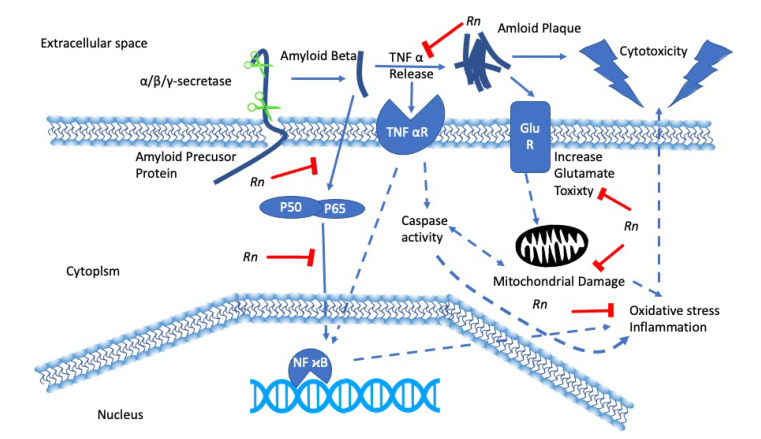
*Rhinacanthus nasutus* (*Rn*) and its protective effects in pathways involved in neurodegenerative diseases. *Rn* inhibits tumor necrosis factor alpha (TNFα) release thus preventing its interaction with the TNFα receptor (TNFαR). *Rn* also prevents nuclear factor kappa-light-chain-enhancer of activated B cells (NF-κB) activation, which in turn leads a reduction in oxidative stress.

Herbal remedies and traditional medicines for the treatment of cognitive decline have been used for centuries, and many have been shown to have positive effects on neurons in a laboratory setting, such as *Curcuma longa* [45], *Mucuna pruriens* [46], and *Bacopa monnieri* [47]. *Rn* is lesser known for its neuroprotective effects; however, there has been much research suggesting that it could be beneficial in the prevention and treatment of neurodegenerative disease [48]. Previous work from our laboratory has shown that *Rn* has potent antioxidant effects in cultured neuronal cell lines subjected to hypoxia [13] and that *Rn* can counteract the cytotoxic effects of glutamate and amyloid-β (Aβ) [14]. We, therefore, hypothesized a reactive oxygen species inhibiting model (Figure 1) that could explain the protective effects of *Rn* [49]. Many other studies have shown that *Rn* possesses potent antioxidant effects, with the crude leaf extract showing similar free radical scavenging properties to the standard Trolox and prevent reactive oxygen species (ROS) induced hemolysis of red blood cells [50,51]. Furthermore, active compounds such as RnC, RnD and RnN being potent superoxide scavengers [52].

There is increasing evidence to suggest that neurodegenerative disease is not restricted to a neurological component; rather, there is a strong interaction with immunological mechanisms in the central nervous system (CNS). In particular, diseases such as Alzheimer’s [53], Parkinson’s [54], multiple sclerosis [55], and ALS [56] have evidence for immunological components. Recent work investigating the neuroprotective effects of RnC (one of the major active compounds found in *Rn*) has shown that RnC is able to attenuate neuroinflammation brought on by LPS, Aβ, and interferon-γ in BV-2 and primary rat glial cells [30]. In particular, this study showed that RnC was able to reduce the release of tumor necrosis factor alpha (TNF-α) and interleukin-6 (IL-6) from BV-2 cells in response to LPS, Aβ, and interferon-γ, as well as reducing NO production and inducible NO synthase (iNOS), IL-1β, C-C Motif Chemokine Ligand 2 (CCL-2), and CCL-5 mRNA levels in primary rat microglial cells. Furthermore, the Chuang et al. 2018 study showed that RnC could prevent damage to primary rat neurons caused by Aβ suggesting that RnC may be a potential in Alzheimer’s disease treatment. The findings from this study are in line with other studies investigating RnC on neuroinflammation [31]. The Chang et al. 2016 study showed that RnC could prevent increases in IL-1β, IL-6, and TNF-α mRNA expression in animals subjected to subarachnoid hemorrhage. This study suggests that RnC may be of benefit to patients who have suffered from delayed ischemic neurologic deficit following an aneurysm by preventing neuroinflammation, and could explain the neuroprotective effects of *Rn* seen in cultured HT-22 cells that were exposed to hypoxic, low glucose conditions and then reperfused with oxygen and glucose [13]. The neuroprotective effects of *Rn* could further be explained by the finding that RnS has acetylcholinesterase activity [20].

Altogether, these studies have shown that *Rn* has neuroprotective properties, with potential for the treatment of Alzheimer’s and aneurysm patients. The crude extract or its active components have both shown potential for reducing neuroinflammation and providing protection against reperfusion injury.

## 4. *Rhinacanthus nasutus* and Cancer

Cancer is the term given to a group of diseases that involve abnormal cell growth, with the potential to spread and invade other parts of the body. The 2014 World Health Organization statistics show that there were approximately 18 million new cases and 9 million deaths worldwide.

In normal healthy cells, there are signals that prevent programmed cell death (apoptosis). There are also signals that lead to apoptosis, for example, when a cell is damaged or has reached the end of its lifespan. There are two classes of genes, which under the right circumstance can cause cancers. The first group is the oncogenes, with roles in the promotion of cell division and cell survival. Well-studied examples include the GTPase Ras [57] tyrosine kinase receptors such as epidermal growth factor receptor (EGFR) [58] and transcription factors such as the *C-Myc* gene [59]. The second class of genes that can lead to cancers are known as tumor suppressor genes. A typical example would be *p53*, which is mutated in 50% of human tumors [60].

The principal treatment methods are a combination of surgery, chemotherapy, and radiotherapy. The latter two do not specifically target cancer cells. Instead, any fast proliferating cell, resulting in several undesirable side effects. Ideal cancer treatment would target a specific property of a cancer cell, preventing its growth and inducing apoptosis without harming healthy cells.

There have been several studies investigating the effects of *Rn* and its constituent compounds on tumor cell growth. The early studies showed that the crude extracts from either the leaves or the roots and some of the extracted compounds could prevent the growth of tumor cell lines (Table 3) such as KB, HEP-2, MCF-7, HepG2, HeLa, SiHa, C-32, LLC, Colon-26, P-388, A-549, HT-29, HL60 and Vero [17,38,61]. Rhinacanthone isolated from the roots inhibited the proliferation of HeLa cells and induced apoptosis via the mitochondrial dependent signaling mechanism [39]. RnC displayed potent cytotoxic and antimetastatic activity against cholangiocarcinoma cells (KKU-M156) by inhibiting invasion regulating focal adhesion kinase (FAK) and interfering with mitogen-activated protein kinase (MAPK) pathway [32]. RnA, isolated from the leaves of *Rn*, was found to be cytotoxic to MCF7 breast cancer cells (50 % inhibition concertation (IC_50_) value of 8.79 μM), and RnN was cytotoxic in NCI-H187 cell line (IC_50_ value of 2.24 μM) and Vero cells (IC_50_ value of 3.00 μM) [20].

## 5. *Rhinacanthus nasutus* and Diabetes

Worldwide, diabetes is regarded as one of the leading causes of death. According to the international diabetes federation, 463 million adults are living with the disease, and the figure is expected to increase by 700 million in 2045 [62]. Diabetes is a cluster of metabolic diseases resulting in a hyperglycemic condition. The increase in blood sugar level may result from impaired insulin secretion and action along with disruption in the normal metabolism of carbohydrates, proteins and lipids, leading to detrimental health effects. Diabetes is also characterized by a rise in fatty acids and triglycerides along with a decline of high-density lipoprotein cholesterol (HDL-C), leading to the deposition of fat and obesity [63]. Hyperglycemia can also increase the level of oxidative stress inside the system, which also contributes to the prevalence of diabetes [10]. Diabetic patients are also prone to suffer from other mild to severe complications like diabetic retinopathy, diabetic nephropathy, diabetic neuropathy, myocardial infarction and multiple organ failure.

Among the several antidiabetic mechanisms, inhibition of α-glucosidase has been reported as the main event in exhibiting the effect, and α-glucosidase inhibitors have been widely prescribed for treating type II diabetes. α-glucosidase is involved in the conversion of starch and disaccharides to simple sugars, thereby increasing the glucose level in circulation [64]. In vitro studies have demonstrated that a rhinacanthin rich extract and its active constituent RnC exhibit noncompetitive inhibition against the α-glucosidase enzyme. In silico studies also showed the positive interaction and binding between RnC and α-glucosidase by hydrogen and hydrophobic bonding [65]. In addition, the rhinacanthin rich extract and RnC also exhibit anti-adipogenetic activity, suggesting that it can also be used effectively against obesity associated-type II diabetes [66].

One of the complex events leading to diabetes is the formation of advanced glycation end products (AGE). AGE’s are the products of the Maillard reaction wherein the proteins are glycated when exposed to sugar molecules, as well as from the degradation products of glucose like methylglyoxal, glycolaldehyde, 3-deoxyglucosone and acts as an important biomarker in diabetes. Elevated levels of AGEs are considered as one of the risk factors for the damage occurring to pancreatic beta cells that secrete insulin [67,68]. Aqueous extract of *Rn* under in vitro conditions has been reported to significantly trap methylglyoxal, thereby inhibiting the formation of advanced glycation end products in a dose-dependent manner [69]. In silico studies also supported the anti-glycation property of RnC by masking lysine and arginine residues in human serum albumin, thereby preventing glycation [52].

**Figure 2:**
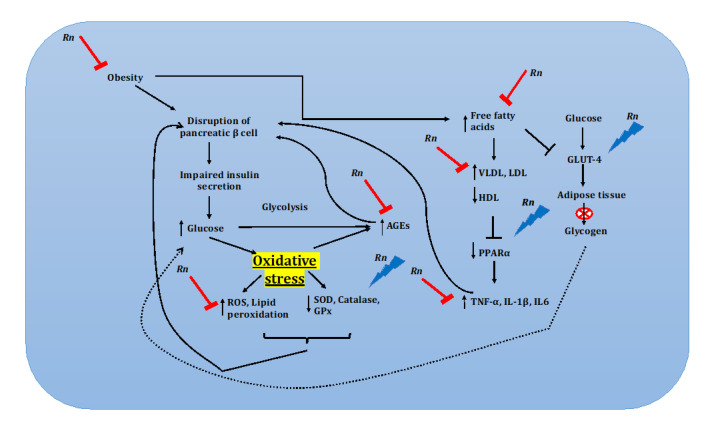
*Rhinacanthus nasutus* (*Rn*) and its beneficial properties regarding diabetes. *Rn* inhibits the production of Advanced glycation end-product (AGEs), prevents the increase in very low-density lipoprotein (VLDL) and low-density lipoprotein, and prevents the decrease in high density lipoprotein (HDL). *Rn* prevents reactive oxygen species (ROS) damage by activating superoxide dismutase (SOD) catalase and glutathione peroxidase (GPx). *Rn* also regulates peroxisome proliferator-activated receptor (PPARα) and glucose transporter type 4 (GLUT-4).

Oxidative stress plays a major role in aggravating the progression of diabetic complications (Figure 2), as the metabolic abnormalities occurring during the process induces the generation of superoxide radicals. This toxic phenomenon further exaggerates the generation of AGE and induces the expression of its receptor [70]. Rhinacanthin-rich extract and RnC were found to scavenge superoxide free radical in cyclic voltammogram analysis [52]. Additionally, they could also reduce the level of lipid peroxidation and improve the levels of antioxidant enzymes, superoxide dismutase (SOD), catalase (CAT) and glutathione peroxidase (GPx), indicating the effect of the plant in reducing oxidative stress [10,21].

Oral administration of methanol extract of Rn at 200 mg/kg body weight in adult male Wistar rats for 30 days reduced the levels of total cholesterol, triglycerides and phospholipids in the liver of diabetic rats to a level like that of non-diabetic control. Further, the extract also reduced the level of low-density lipoprotein-cholesterol (LDL-C) and very-low-density lipoprotein-cholesterol (VLDL-C) in the diabetic rats apart from improving the levels of HDL-C, thereby exhibiting hypolipidemic activity in diabetic rats. More importantly, the plant did not exhibit any hypoglycemic activity in normal rats indicating that it was non-toxic to normal rats [63]. Impaired lipoprotein and lipid metabolism are a characteristic feature of type II diabetes. Pathological changes include increased hepatic secretion and impaired clearance of VLDL [71]. One of the key players in lipid metabolism is insulin, which helps in the storage of triacylglycerols (TAG) and reduces the generation of VLDL by declining the level of non-esterified fatty acids in circulation. Similar to *Rn* extract, oral administration of rhinacanthin-rich extract and RnC significantly normalized the water and food intake in diabetic rats, along with the bodyweight, without causing any significant changes in the normal rats. It also decreased the hyperglycemic levels and destruction of beta cells in the pancreas and increased the serum insulin gradually in diabetic rats. The elevated levels of aspartate aminotransferase (AST) and alanine aminotransferase (ALT) along with blood urea nitrogen (BUN) and creatinine in diabetic rats were significantly reduced by the extract treatment indicating the normalization of the liver as well as kidneys functioning in diabetic rats [72]. Similar results were also reported by Rao et al. 2013, where the key markers of liver functioning were normalized in diabetic rats upon treatment with the plant extract [73]. Further, RnC also modulated the levels of fasting blood glucose (FBG) and glycated hemoglobin (HbA1c) in diabetic rats. HbA1c is a form of hemoglobin that is in bonding with sugar moiety and is formed when there is an excessive circulation of glucose in the bloodstream. Moreover, RnC could also reduce the insulin resistance index, modulate antioxidant enzymes, and prevent the shrinking of the islet of Langerhans in diabetic rats [74]. The reported health benefits of the plant extract against diabetic condition could probably be mediated by the activity of cytosolic and mitochondrial enzymes. The level of cytosolic enzymes like glucose-6-phosphate dehydrogenase (G6PDH), glutamate dehydrogenase (GDH) and mitochondrial enzyme succinate dehydrogenase (SDH) is reduced in individuals with diabetes, where G6PDH plays a vital role in antioxidant defense against oxidative stress by the production of NADPH [75] while GDH and SDH are involved in insulin secretion [76,77]. Administration of *Rn* extract significantly restored the levels of these enzymes, thereby ameliorating the diabetic conditions in diabetic rats [73].

Pioneering studies suggest the relationship between chronic inflammation and the pathophysiology of diabetes, and immunomodulatory drugs have been advised in the treatment of individuals with type II diabetes [78,79]. Free fatty acids and cholesterol induce inflammation in diabetic individuals and subsequently induces the release of proinflammatory cytokines and chemokines mediated by NFκB [80,81]. Rhinacanthins-rich extract and RnC administration reduced the levels of free fatty acids and cholesterol, thereby attenuated proinflammatory cytokines TNF-α, IL-1β, and IL6 in diabetic rats. In addition, the renal concentrations of caspase-3 and cytochrome C were also reduced in the kidneys of diabetic rats. Diabetes induced histological changes in the kidney such as thickening of the basement membrane of the renal tubules and necrosis of renal tubules were also reduced by the treatment of extract, indicating that the plant extracts can also be used as immunomodulatory agents in alleviating the complications of diabetes [21].

Other than reducing the glucose, triglyceride and cholesterol level, administration of *Rn* can also stimulate the expression of peroxisome proliferator-activated receptor (PPARα), fat adiponectin, glucose transporter type 4 (GLUT4) protein expression [82]. Expression of PPARα and GLUT4 are highly downregulated in individuals affected with diabetes, and both play a major role in the regulation of glucose, lipid metabolism and transports glucose from the circulation into insulin-sensitive cells, respectively [83,84]. PPAR agonists were also found to improve against insulin resistance and to reduce the level of TG and glucose [85]. The stimulation of these vital proteins upon *Rn* extract administration could be the reason behind its antidiabetic effect. Further, the pharmacokinetic and toxicity profile of RnC by in silico approach suggests that the phytochemical is devoid of carcinogenicity, mutagenicity and non-toxic, suggesting it is safe to use as an antidiabetic drug [72].

## 6. Antibacterial, Anti-Fungal, and Anti-Viral Activities of *Rhinacanthus nasutus*

Since the discovery of penicillin and its derivatives nearly a century ago, the world’s population has grown, and deaths caused by bacterial infections has fallen dramatically. However, in recent years inappropriate and overuse has resulted in the emergence of antibiotic-resistant strains of bacteria, resulting in the need to find novel antibiotics. Extracts of *Rn* have been tested against a range of bacterial strains, and it has been shown to have effective antibiotic activities against Gram-positive strains, including *Bacillus cereus, Bacillus globigii, Bacillus subtilis,* and *Staphylococcus aureus*. However, it was not effective against Gram-negative strains such as *Salmonella typhi, Pseudomonas aeruginosa* or *Escherichia coli* [86], presumably due to the difference in the cell wall made up of Gram-positive and negative bacteria. The rhinacanthin-rich ethanol extract of *Rn* leaves has been shown to have activity against the ulcer and cancer-causing Gram-negative bacteria, *Helicobacter pylori*, with a minimum inhibitory concentration (MIC_50_) of 0.5 mg/mL [4]. Further studies have been carried out on RnC extracted from *Rn*, showing that the MIC_50_ for RnC against *S. mutans*, *P. acnes*, *S. aureus* and *S. epidermidis* are two, eight, two and eight µg/mL, respectively [4]. Studies, including RnN and RnQ, showed that the MIC_50_ ranged from 4.9 to 19.53 µg/mL for Gram-positive bacteria but had no anti-biotic effects on Gram-negative bacteria. Interestingly RnN was shown to be more potent than gentamycin in the inhibition of the Gram-positive bacteria [87].

Traditionally the extracts from *Rn* have been used for the treatment of skin infections with fungi, such as ringworm and tinea cruris [88]. *Rn* has been shown to be effective against a range of clinically relevant fungi, including *C. albicans* (of the most common hospital-acquired infections), *T. mentagrophytes* (a common cause of ringworm), *C. tropicalis* (a common cause of oral thrush, and particular risk to HIV patients) and *C. parapsilosis* (Cause of endocarditis in patients with prosthetic valves) [86].

Post-2019 and the Severe acute respiratory syndrome coronavirus 2 (SARS-CoV-2) pandemic that has spread around the world at an alarming rate, it has become clear that there is a need for new and effective antiviral drugs. *Rn* and its constitutive compounds have been shown to be effective against various viruses, including Herpes [22,89,90] and Influenza [23]. Recent research has shown that compounds isolated from the aerial parts of *Rn* have neuraminidase inhibitory activity. Neuraminidase is a target on the surface of the influenza virus that plays an important role in the entry of the virus into the cell. It has been postulated that neuraminidase inhibitors may be of benefit in the treatment of SARS-CoV-2 [91]. However, any *Rn* compound would still need to be tested clinically, and there are already several neuraminidase inhibitors that have been tested for safety and would be ahead of any *Rn* compound with regards to clinical testing against SARS-CoV-2.

## 7. Conclusions

*Rn* is a medicinal herb that contains several compounds that are potentially useful medically. RnC is of interest, particularly in the treatment of diabetes and neurodegeneration, where preclinical studies have shown it to be effective in controlling diabetes by inhibiting α-glucosidase and preventing neurodegeneration caused by Aβ. There is strong evidence for the effectiveness of RnC for it to be entered in clinical trials for diabetes and dementia. Other compounds extracted from *Rn* appear to be effective against cancer cell lines. Although these studies are less advanced and the mechanisms yet to be fully understood, there is still potential for *Rn* in cancer treatment.

## Figures and Tables

**Table 1:** List of common/regional names of *Rhinacanthus nasutus* (L.) Kurz.

Synonyms	
*Rhinacanthus nasutus* (L.) Kurz	
*Rhinacanthus communis* Nees	
*Rhinacanthus osmospermus* Bojer ex Nees	
*Justicia dichotoma* Rottler *	
*Justicia nasuta* L.	
*Justicia macilenta* E. Mey. *	
*Justicia rottleriana* Wall. *	
*Justicia silvatica* Nees *	
*Justicia sylvatica* Vahl *	
*Pseuderanthemum connatum* Lindau	
*Dianthera paniculata* Lour.	
**Common Names**	**Common Name Origin**
জূঈপান Juipana	Bengali
Dainty Spurs, Snake jasmine White Crane flower	English
पालकजूही Palakjuhi, जूहीपानी Juhipani	Hindi
ನಾಗಮಲ್ಲಿಗೆ Nagamallige, Doddapatike	Kannada
Dadmari	Konkani
Puzhukkolli Nagamulla Purukolli Orukaalmudanthi Vellakkurunji Nagamulla (നാഗമുല്ല) Pushpakedal	Malayalam
गजकर्णी Gajkarni	Marathi
Yudhikaparni, Yoodhikaparni	Sanskrit
Uragamalli Nagamalli (நாகமல்லீ)	Tamil
Nagamalle (నాగమల్లె)	Telugu
Palakjuhi	Urdu
Thong Phan chang (ทองพันชั่ง), Yaa man kai (หญ้ามันไก่) (ตะ ซิ ชี ซี) Ta si chi si (กะเหรี่ยงสะกอ)	Thai

* Name is unresolved, but some data suggest that it is synonymous with *Rhinacanthus nasutus* (L.) Kurz (*Rn*) (checked via the plant list [15].

**Table 2:** Structures and activities of compounds found specifically in *Rhinacanthus nasutus* extracts.

Name	Structure	Reported Activities
2-Hydroxy-1,4-naphthoquinone	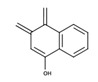	An interconversion of pyridine nucleotides to combat the effects of oxidative stress [26]
3,4-dihydro-3,3-dimethyl-2H-naphtho[2,3-b] pyran-5,10-dione	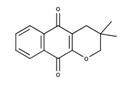	Antitumor activity [27]
*Rhinacanthin A*	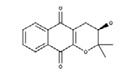	Inhibited the growth of *S. aureus* with an inhibition zone of 16 mm (25 mm disk) [28]
*Rhinacanthin B*	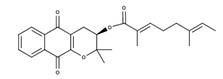	Inhibition of cytochrome enzymes CYP6AA3 and CYP6P7 in vitro [29]
*Rhinacanthin C*	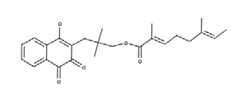	Prevented Aβ-induced toxicity in rat hippocampal neurons and attenuated lipopolysaccharides (LPS)-activated nitric oxide (NO) production, inducible NO synthase (iNOS) expression, and nuclear factor kappa-light-chain-enhancer of activated B cells (NF-κB) signaling in rat glia [30] ;Exhibited neuroprotective effect by reducing cleaved caspase-3- and caspase-9a-related apoptosis and anti-inflammatory effect by decreasing High mobility group box 1 (HMGB-1) mRNA and protein expression [31]; Inhibits cholangiocarcinoma cell migration and invasion by decreasing matrix metalloproteinase-2 (MMP-2), Urokinase-Type Plasminogen Activator (uPA), focal adhesion kinase (FAK) and mitogen-activated protein kinase (MAPK) pathways, along with inhibition of cell migration and antiproliferative effects [32]
*Rhinacanthin D*	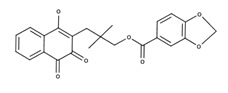	Inhibitory activity against cytomegalovirus [22]; antiviral activities against influenza virus A/PR/8/34 (PR8), Anti-Human Rhinovirus 1B (HRV1B), and Coxsackievirus B3 (CVB3)-infected Vero cells [24]
*Rhinacanthin E*	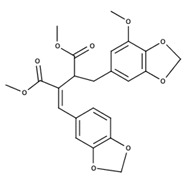	Antiviral activity against influenza virus type A [23]
*Rhinacanthin F*	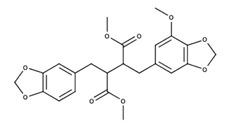	Antiviral activity against influenza virus type A [30]
*Rhinacanthin G*	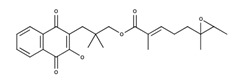	Inhibition of cytochrome enzyme CYP6P7 in vitro [29]
*Rhinacanthin M*	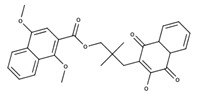	Inhibition of cancer cell lines KB, HeLa, and HepG_2_ with IC_50_ values of 1.5, 3.0 and 4.6 µM, respectively [33]
*Rhinacanthin N*	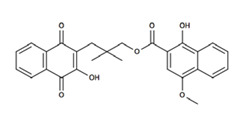	Rhinacanthin-N caused G2/M arrest of HeLaS3 cells after 24 h incubation and increased the proportion of sub-G1 hypodiploid cells, apoptotic cells [34]; Antimetastatic activity as it inhibited the metastatic pulmonary colonization of the melanoma cells in C57BL/6 male mice [35]; Antiproliferative activity against HeLaS3 cells and suppressed tumor growth in vivo [36]
*Rhinacanthin O*	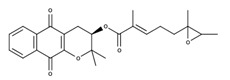	Unknown activities
*Rhinacanthin Q*	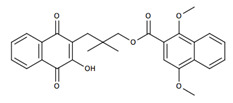	Induction of apoptosis in tumor cells that may be associated with the activation of the caspase-3 pathway [34]; Antiviral activities against PR8, HRV1B, and CVB3-infected Vero cells [24]
*Rhinacasutone*	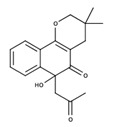	Unknown activities
*Heliobuphthalmin*	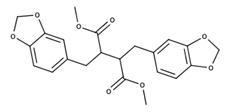	Strong inducer of apoptosis in HuH-7 cells [37]; High antineoplasic activities against the classical (multi-drug-resistant) MDR subline derived from gastric carcinoma [38]
*Rhinacanthone*	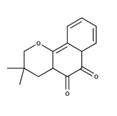	Rhinacanthone-induced apoptosis in HeLa cells is mediated primarily through the mitochondrial-dependent signaling pathway as it inhibited proliferation of HeLa cells along with chromatin condensation, internucleosomal DNA fragmentation, increase in the proportion of sub G(1) apoptotic cells, and externalization of annexin-V. Increase in the level of Bax and a decrease in the level of Bcl-2 and activation of caspase 3 and 9 [39]; Inhibited tumor cell growth in Dalton’s ascitic lymphoma (DAL) in Swiss albino mice [40]

**Table 3:** IC_50_ values for various extracts and isolated compounds from *Rn* in a range of cancer cell lines. Data extracted from Wu et al. 1998 [17], Siripong et al. 2006 [61] and Siripong et al. 2009 [39].

Cell Line	Chloroform Extract µg/mL	Methanol Extract µg/mL	RnC µM	RnD µM	RnG µM	RnO µM	RnM µM	RnN µM	RnQ µM	Rhinacanthone µM
KB	0.55	3.9	0.46	0.47	4.7	5.5	2.6	0.33	1.4	3.8
Hep-2	0.3	4.0	0.8	7.6	3.3	3.7	6.1	1.2	3.6	4.4
MCF-7	0.8	20.0	0.88	14.7	8.7	8.1	8.9	2.6	10.6	4.9
HepG2	0.95	8.5	0.41	1.9	1.2	6.5	3.8	0.37	3.0	4.9
HeLa	0.39	4.4	0.29	0.49	4.7	6.1	4.3	0.87	3.8	4.2
SiHa	1.5	21.0	0.49	6.6	18.8	7.4	39.1	3.9	6.1	2.9
C-32	5.0	30.0	9.8	14.7	16.4	30.7	37.0	39.1	8.4	2.1
LLC	1.8	40	0.98	6.1	6.1	8.2	54.4	5.4	8.3	2.9
Colon-26	0.3	5.0	0.44	2.3	1.5	6.6	5.4	1.1	1.1	3.4
P-388	0.3	7	1.5	9.3	3.3	8.9	8.1	3.7	10.3	4.4
Vero	0.2	2.5	11.0	34.3	16.4	12.3	36.1	12.7	41.1	4.2
